# Hot or Not: Response Inhibition Reduces the Hedonic Value and Motivational Incentive of Sexual Stimuli

**DOI:** 10.3389/fpsyg.2012.00575

**Published:** 2012-12-26

**Authors:** Anne E. Ferrey, Alexandra Frischen, Mark J. Fenske

**Affiliations:** ^1^Department of Psychology, University of GuelphGuelph, ON, Canada

**Keywords:** response inhibition, affective devaluation, incentive salience, motivation, sexual attractiveness

## Abstract

The motivational incentive of reward-related stimuli can become so salient that it drives behavior at the cost of other needs. Here we show that response inhibition applied during a Go/No-go task not only impacts hedonic evaluations but also reduces the behavioral incentive of motivationally relevant stimuli. We first examined the impact of response inhibition on the hedonic value of sex stimuli associated with strong behavioral-approach responses (Experiment 1). Sexually appealing and non-appealing images were both rated as less attractive when previously encountered as No-go (inhibited) than as Go (non-inhibited) items. We then discovered that inhibition reduces the motivational incentive of sexual appealing stimuli (Experiment 2). Prior Go/No-go status affected the number of key-presses by heterosexual males to view erotic-female (sexually appealing) but not erotic-male or scrambled-control (non-appealing) images. These findings may provide a foundation for developing inhibition-based interventions to reduce the hedonic value and motivational incentive of stimuli associated with disorders of self-control.

## Introduction

Stimuli that are ignored or from which a motor response is withheld subsequently receive more negative affective ratings than novel items or the targets of attention/response (e.g., Raymond et al., 2003; for reviews, see Fenske and Raymond, 2006; Raymond, 2009). This form of affective devaluation is robust, having been found with several classes of visual stimuli in a range of experimental paradigms thought to involve attentional- or response-related inhibition (e.g., Fenske et al., 2004, 2005; Kihara et al., 2011; Frischen et al., 2012). This close link between inhibition and aversive response has been taken as evidence that mechanisms of cognitive control and emotion work together to ensure that previously distracting stimuli are effectively avoided in future encounters (e.g., Fenske and Raymond, 2006; Frischen et al., 2012).

Several studies have been conducted to reveal the fundamental characteristics of this *inhibitory devaluation* effect. For example, it increases with distractor salience or the extent to which a distractor interferes with target selection (Raymond et al., 2005; Frischen et al., 2012). Inhibitory devaluation also appears to be mediated by visual working memory (Goolsby et al., 2009a), and can generalize to influence evaluations of stimuli with features similar to those of a prior distractor (Raymond et al., 2003; Goolsby et al., 2009b). Yet, despite all that is now known about the effect, surprisingly little consideration has been given to the potential applications or clinical significance of inhibitory devaluation.

To address this void, we examined some critical issues related to the potential usefulness of response inhibition tasks for assisting individuals with disorders of self-control – sexual compulsivity, drug addiction, etc. The first issue we addressed (Experiment 1) concerns the type of stimuli that feature heavily in disorders of self-control, which are predominantly those associated with a strong behavioral-approach response. While prior work has established that cognitive inhibition has negative affective consequences for a variety of stimuli – abstract art-like patterns, face images, real-world objects, complex scenes, and even emotionally salient stimuli (e.g., Fenske et al., 2005; Griffiths and Mitchell, 2008; Kiss et al., 2008; Veling et al., 2008; Frischen et al., 2012) – it is possible that the strong approach-related response evoked by more motivationally relevant stimuli may prevent the formation of inhibition-related negative affect. We utilized sexual images to examine this issue, given the well-known capacity of sex stimuli to, even in healthy individuals, be rewarding (Bray and O’Doherty, 2007), elicit strong behavioral-approach responses (Aharon et al., 2001), and impair the disengagement of attention (Israel and Strassberg, 2009). In sum, our first objective was to establish whether response inhibition has negative consequences for affective ratings of sexually relevant stimuli. We did this by presenting erotic images of participants’ preferred and non-preferred-sex in a response inhibition (Go/No-go) task and then obtaining affective ratings of sexual attractiveness for previously inhibited images (prior No-go trials) and non-inhibited images (prior Go trials).

Our second objective (Experiment 2) was to assess whether the effects of response inhibition extend beyond subjective emotional impressions (i.e., affective ratings) of a stimulus to also impact behavioral outcomes. Prior work (e.g., Berridge and Robinson, 2003; Berridge et al., 2009; Dai et al., 2010) suggests that how much an individual may “like” something (e.g., subjective evaluation of hedonic pleasure) does not always correspond with how much they may “want” that thing (e.g., the motivational incentive to seek and obtain the stimulus). And from a practical perspective, the potential usefulness of a task for helping individuals control their behavior critically depends on whether the task can actually influence behavior. Therefore, the novelty of our investigation, beyond the use of sex stimuli in a standard inhibitory devaluation paradigm in Experiment 1, is the inclusion in Experiment 2 of a key-press task to operationalize the amount of time and behavioral effort participants were willing to expend to view erotic images. This allowed us to directly assess whether prior inhibition, in addition to lowering ratings of hedonic value, also reduces the behavioral incentive to seek and obtain otherwise-appealing items.

A striking feature of many self-control disorders is the amount of time and effort individuals will expend to obtain a reward-related outcome, despite negative consequences (e.g., Hyman and Malenka, 2001). If the affective and motivational incentive of objects associated with reward-related pursuits can be reduced through cognitive inhibition, then this may effectively reduce the potential of such stimuli to capture attention (Field and Cox, 2008) and drive behavior (Berridge and Robinson, 1995) at the cost of other physical and psychological needs. The long-term objective of this type of work is therefore to develop and test the effectiveness of inhibition-based interventions in specific clinical populations. However, because the feasibility of such efforts depends on basic characteristics of the inhibitory devaluation effect, we begin here by focusing on these issues in healthy individuals.

Interventions aimed at decreasing maladaptive responses to motivationally salient stimuli often focus on enhancing willpower or cognitive control over impulses. Other approaches try to reduce the motivational incentive of the stimuli themselves; for example, by administering psychopharmacological compounds that block the rewarding effects of drugs of abuse (Volpicelli et al., 1992). However, if it can be shown that an inhibitory task can selectively reduce the motivational incentive of highly salient stimuli, as well as their subjective hedonic evaluation, this would lay a solid foundation for the future development of simple computer-based interventions for compulsive viewing of erotic stimuli and other disorders of self-control.

## Experiment 1

The aim of Experiment 1 was to assess the impact of response inhibition on hedonic ratings of sexual attractiveness for erotic images of participants’ preferred and non-preferred-sex. We asked participants who were strongly attracted to either males or females to complete a Go/No-go task that required them to refrain from responding on half the trials (“No-go”). Because we expect that inhibition will reduce the hedonic value of approach-related stimuli (i.e., preferred-sex images), we predicted that these items will receive lower subjective ratings of sexual attractiveness after being presented on No-go trials, compared to those presented on Go trials. We also anticipated inhibition-related devaluation of non-preferred images based on prior findings that inhibition has negative consequences for both positively valenced and negatively valenced stimuli (Frischen et al., 2012).

### Methods

Fifty participants with normal or corrected-to-normal vision were recruited from the University of Guelph undergraduate participant pool in exchange for course credit or $10. Data from seven participants were excluded because of low accuracy on No-go trials (>2 SD below the reported mean). This exclusion criterion was based on evidence that poor No-go performance can reflect impairments in attention and the inability to exert inhibition (see, e.g., Aron and Poldrack, 2005). Data from an additional six participants were excluded because they indicated equal attraction to males and females or for failing to complete the entire task as directed. Only individuals strongly attracted to males or females were included because our hypotheses specifically related to the impact of inhibition on sexually relevant stimuli. Results are reported for the remaining *n* = 37 participants (mean age 22.2 years, SD = 9.2; 24 females). All of the 13 males within this sample reported being strongly attracted to females, while all of the 24 females reported being strongly attracted to males.

**Figure 1:**
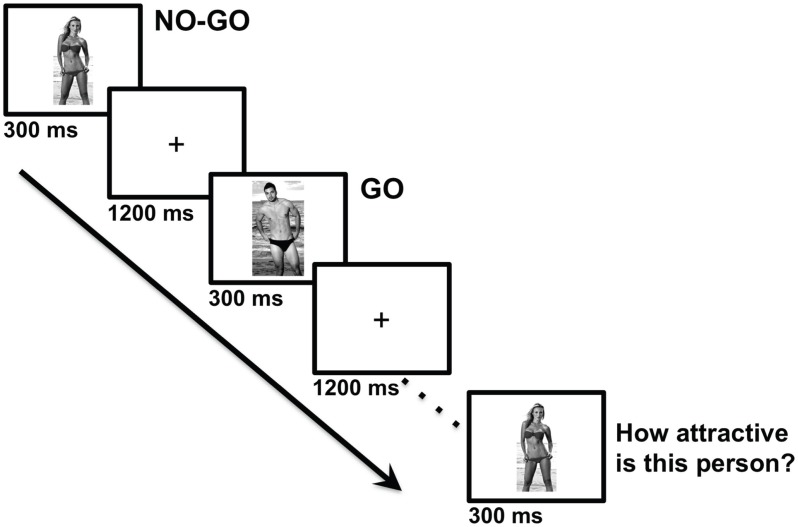
**Example of the stimulus sequence in the Go/No-go task of Experiment 1**. In this example, attractive male images are “Go” stimuli that require a speeded key-press response, and attractive female images are “No-go” stimuli that require the response to be withheld. After every 12 Go/No-go trials, the same stimuli are affectively evaluated. Photo credits Evan Balbier and Elmo Love.

Stimuli were 192 digital color photographs of attractive males and females. No nudity was shown, but images were selected for their explicit sexual appeal (see Figure 1). Half of the images were males and half were females, and both sets contained an equal number of light-haired and dark-haired people. Each image, comprising 10.6 × 16.3 visual angle, was presented for 300 ms on a light gray background at the center of the screen. A central black fixation cross was displayed for 1200 ms between image presentations. Stimulus presentation and behavioral response collection were controlled by E-Prime 2.0 software (Psychology Software Tools, Pittsburgh, PA, USA) running on an Intel Core 2 Duo computer with a 50.8 cm LCD monitor (resolution: 1680 × 1050 pixels).

All procedures were approved by the Research Ethics Board at the University of Guelph. The experimental paradigm was based on the fully factorial within-subjects design used by Kiss et al. (2008) which combined Go/No-go and affective-evaluation tasks. Participants performed 16 blocks, each comprising 12 Go/No-go trials followed by 12 evaluation trials. The Go/No-go trials required participants to press the spacebar with both index fingers as quickly as possible whenever the displayed person had dark hair (or light hair; this Go cue switched once halfway through the session and its order was counterbalanced across participants) and to otherwise refrain from responding. Images were randomly selected with the constraint that each set of 12 trials comprised equiprobable factorial combinations of sex and hair color. In the evaluation trials, participants judged the attractiveness of the same 12 images that were presented in the same order for 300 ms each followed by a 2700 ms blank interval. Images were rated on a four-point scale from 1 (“Not at all attractive”) to 4 (“Very attractive”) by pressing the corresponding numeric key on a standard keyboard. Participants were instructed to rate how attractive they personally found each person, rather than estimate objective attractiveness. After completion of these tasks, participants indicated the sex to which they are most attracted on a 9-point scale, anchored by 1 (“Attracted exclusively to men”), 5 (“Attracted to both sexes equally”), and 9 (“Attracted exclusively to women”).

### Results and discussion

Only ratings of images associated with a correct Go/No-go response were included in our analyses. This eliminated 13.8% of No-go trials and 10.4% of Go trials. The remaining data were then analyzed with 2 (Sexual-Relevance: Preferred-sex vs. Non-preferred) × 2 (Response-status: Go vs. No-go) repeated-measures ANOVAs.

**Figure 2:**
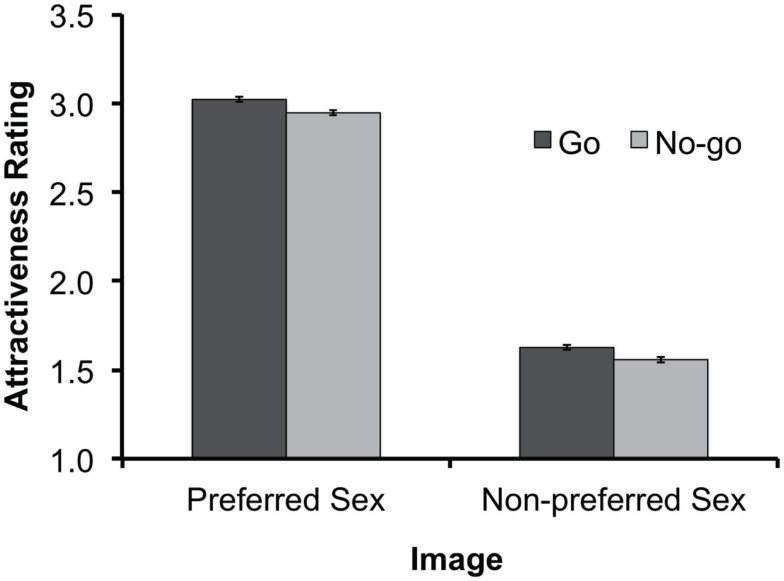
**Results of Experiment 1: mean hedonic-evaluation scores for each response type (“Go” vs. “No-go”) and for images of the preferred and non-preferred-sex**. Evaluation scores ranged from 1 to 4, with larger values representing more positive hedonic ratings. Error bars represent standard errors of the means based on Loftus and Masson’s (1994) method for within-subjects designs.

Participants rated Preferred-sex images (*M* = 2.99) as significantly more attractive than Non-preferred images (*M* = 1.59), *F*(1, 36) = 114.47, *p* < 0.001, confirming their sexually appealing nature. Furthermore, participants rated “No-go” images as significantly less attractive than “Go” images, *F*(1, 36) = 6.47, *p* < 0.05; this was the case for both the Preferred-sex [*t*(36) = 2.06, *p* < 0.05] and the Non-preferred conditions [*t*(36) = 2.43, *p* < 0.05; see Figure 2]. There was no significant interaction between these effects (*F* < 1). Despite our decision to exclude individuals with poor accuracy on No-go trials, it should be noted that repeating our statistical analyses while including these participants produced equivalent results: attractiveness ratings of prior “No-go” stimuli were significantly lower than those of prior “Go” stimuli for both preferred-sex images [*t*(43) = 2.51, *p* < 0.05] and non-preferred images [*t*(43) = 3.33, *p* < 0.01]. Both sexually appealing and non-appealing stimuli become hedonically devalued after being associated with response inhibition.

Based on prior reports that visual sex stimuli may provide stronger motivational incentive for men than women – e.g., males look more exclusively at preferred-sex vs. non-preferred images (Lykins et al., 2008) and are more likely to spend greater amounts of time online viewing sexually explicit images (e.g., Cooper et al., 2000) than women – we re-analyzed our data using a mixed ANOVA that included Participant-sex (Male vs. Female) as a between-subjects factor. Importantly, neither the Participant-sex by Response-status interaction, nor the higher order interaction between these factors and Sexual-relevance, were significant (all *F*s < 1), suggesting that the sex of the participant does not affect the magnitude of inhibitory devaluation of sex stimuli. However, consistent with prior evidence that men respond more strongly to visual sexual stimuli, there was a significant Participant-sex by Sexual-relevance interaction, *F*(1, 35) = 14.84, *p* < 0.001. This was characterized by a greater response to Preferred-sex vs. Non-preferred images for male participants than for female participants: males indicated significantly greater sexual attraction to Preferred-sex images (*M* = 3.18) than that indicated by females (*M* = 2.78), *t*(35) = 2.50, *p* < 0.05, and significantly less sexual attraction to Non-preferred images (*M* = 1.20) than that indicated by females (*M* = 1.69), *t*(35) = −3.38, *p* < 0.01.

## Experiment 2

The results of Experiment 1 established that inhibiting motor responses toward sex stimuli decreases their subjective hedonic value. In Experiment 2, we tested whether prior inhibition, in addition to lowering hedonic ratings, also reduces the behavioral incentive to seek and obtain otherwise-appealing items. We combined a Go/No-go task with a subsequent progressive-ratio key-press task (Hodos, 1961). The key-press task operationalizes the amount of time and effort participants are willing to expend to view an image as a measure of its motivational incentive (Aharon et al., 2001; Levy et al., 2008). We expect that inhibition will decrease the motivational incentive of sexual images; thus, participants should execute fewer key-presses to see images of the sort previously encountered on No-go trials compared to those seen on prior Go trials.

A noteworthy feature of Experiment 2 is that only male participants were tested. This decision was based in part on the potential for our findings to inform future clinical applications, given evidence that that men spend more time seeking and viewing sexually explicit images and report greater corresponding levels of impulsivity, compulsivity, and other negative effects than women (e.g., Wetterneck et al., 2012). The rationale was also based on our finding that males, when compared to female participants, showed a stronger hedonic response to preferred-sex vs. non-preferred images in Experiment 1, along with corresponding evidence that visual sex stimuli tend to provide the greatest behavioral-approach incentive for males (for review, see Rupp and Wallen, 2008). A male-only sample should therefore provide a more stringent test of our hypothesis that response inhibition can reduce the motivational incentive of stimuli that otherwise elicit a strong behavioral-approach response.

The potential clinical significance of our findings from Experiment 2 depends not on whether prior inhibition can reduce the amount of time and effort participants are willing to expend to view a set of exact images, *per se*, but on whether it reduces the motivational incentive to view other images of the same type. Prior work has demonstrated that the affective consequences of inhibition can indeed generalize to influence evaluations of other stimuli, as long as they are from the same category or otherwise share the same features as previously inhibited items (Raymond et al., 2003; Goolsby et al., 2009b). Presenting images in the subsequent progressive-ratio key-press task that are novel but from the same categories as those that previously appeared as either No-go (inhibited) or Go (non-inhibited) stimuli therefore allowed a stringent test of our hypothesis that inhibition can reduce the motivational incentive of stimuli that otherwise elicit a strong behavioral-approach response. Note, however, that while we were particularly interested in the effect of response inhibition on the motivational incentive of sexually appealing images, our Experiment 1 finding that prior inhibition resulted in lower hedonic ratings of both preferred-sex and non-preferred image suggests that motivational incentive may likewise be reduced both when response inhibition is applied to preferred-sex images and when applied to non-preferred images.

### Methods

Eighty-five new participants were recruited in the same manner as Experiment 1, with the exception that only male participants were invited to participate. Thirteen participants were excluded due to low accuracy on No-go trials (>2 SD below the mean) using the same criteria as Experiment 1. An additional nine participants were excluded because they were not strongly attracted to females or for failing to complete the task as directed. Results are reported for the remaining *n* = 63 participants (mean age 19.9 years, SD = 2.98). Including individuals reporting only strong attraction to females allowed greater comparability with the results of the male participants from Experiment 1, who also happened to report only strong attraction to females.

Stimuli used for the Go/No-go task were identical to those in Experiment 1. Stimuli used in the progressive-ratio key-press task were a separate set of similar digitized color photographs of attractive females and males. In addition to attractive-female and attractive-male images, scrambled versions of these stimuli were created by dividing each image into a grid of 676 equally sized boxes and randomly varying the locations of the boxes within the grid. Including these scrambled-female and scrambled-male images in the key-press task provided a non-sexual baseline of viewing options that were matched with the sexually relevant options in terms of low-level visual information that might otherwise account for differences in viewing choices (e.g., image coloration; McManus et al., 1981).

All procedures were approved by the Research Ethics Board at the University of Guelph. Participants performed a task identical to that of Experiment 1, except that the No-go stimuli for each group of participants were consistently either erotic-female images or erotic-male images. This resulted in Response-status (No-go vs. Go) being a between-groups factor in Experiment 2. Afterward, participants performed a progressive-ratio key-press task to measure the motivational incentive of the stimuli. In this task, participants fixated a cross in the center of a blank screen, having been instructed to press any of the numerical keys from 1 to 4 in order to see a 1-s presentation of one of four different image types assigned to a given key: erotic-female images, erotic-male images, scrambled-female images, and scrambled-male images. The keys were on a progressive-ratio schedule such that twice as many key-presses were required for each successive view of the sort of image associated with that key. Thus, if a participant pressed a key, saw a corresponding image, and then wanted to view another image of that type, they would then have to make twice as many key-presses to be rewarded with another image of that type. Participants responded, pressing any key in any combination, until they had seen 10 images (from any combination of image types) and then moved on to a new trial with a new randomly determined mapping of key to image type. Thus, at the beginning of each trial, participants did not know what type of image would be revealed by pressing a specific key until they had actually pressed it. Participants performed eight trials of the progressive-ratio key-press task. Afterward, they filled out a demographic questionnaire, which included an indication of their sexual attraction to males and females, as in Experiment 1.

### Results and discussion

For hedonic ratings, 5.9% of No-go trials and 3.9% of Go trials were excluded from analysis due to incorrect performance. Because each participant experienced only one particular combination of Response-status and Sexual-relevance conditions (i.e., Preferred-sex images on No-go trials and Non-preferred images on Go trials, or vice versa) the impact of response inhibition on hedonic ratings of erotic images had to be assessed between-groups. We did this using a two-way ANOVA that treated both Sexual-relevance (Preferred-sex vs. Non-preferred) and Response-status (Go vs. No-go) as between-subjects factors.

**Figure 3:**
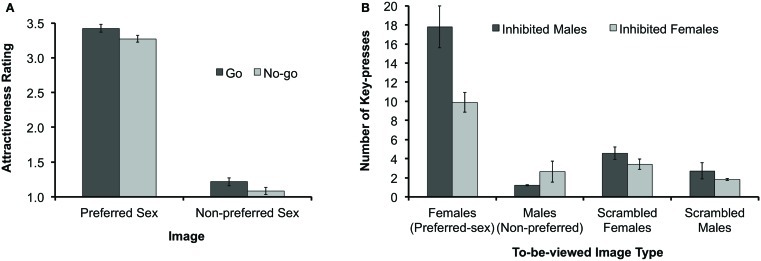
**Results of Experiment 2**. **(A)** Mean hedonic-evaluation scores for each response type (“Go” vs. “No-go”) and for Preferred-sex and Non-preferred images. Evaluation scores ranged from 1 to 4, with larger values representing more positive hedonic ratings. **(B)** Number of key-presses to see attractive female images, attractive male images, and scrambled versions of these images, for participants who had previously inhibited either attractive female or attractive male images. Error bars represent standard errors of the means.

As in Experiment 1, participants rated Preferred-sex images (*M* = 3.35, SD = 0.33) as significantly more attractive than Non-preferred images (*M* = 1.15, SD = 0.28), *F*(1, 122) = 1130.43, *p* < 0.001. Participants also rated prior “No-go” images (*M* = 2.20, SD = 1.14) as significantly less attractive than prior “Go” images (*M* = 2.30, SD = 1.16), *F*(1, 122) = 5.18, *p* < 0.05 (see Figure 3A). There was no significant interaction between these effects (*F* < 1). Taken together, these results provide an important replication of our Experiment 1 finding that sexually relevant stimuli become hedonically devalued after being associated with response inhibition. However, the effect of response inhibition on subsequent ratings was not significant when our affective-rating analyses were repeated while including the individuals with poor accuracy on No-go trials (*p* > 0.6). This suggests that the ability to observe inhibitory devaluation may be impacted by impairments in inhibitory control, to the extent that such impairments are reflected by poor No-go performance (e.g., Aron and Poldrack, 2005).

Regarding our primary hypothesis about the impact of inhibition on the motivational incentive of sexually appealing images, response-status did modulate the frequency of key-pressing, particularly for intact Preferred-sex images. A 2 (Sexual-relevance) × 2 (Response-status) mixed-factors ANOVA of responding for intact images revealed significant effects of both Sexual-relevance [*F*(1, 61) = 86.09, *p* < 0.001] and Response-status [*F*(1, 61) = 5.63, *p* < 0.05] as well as a significant interaction between these effects, *F*(1, 61) = 13.06, *p* < 0.01. Planned contrasts revealed that participants who had inhibited female stimuli subsequently made significantly fewer key-presses in order to see female images compared to participants for whom female stimuli were the targets of response, *t*(61) = 3.28, *p* < 0.01 (see Figure 3B). There were no significant differences in key-pressing for intact-male images or any of the scrambled images (all *p*s > 0.17). Unlike our hedonic-rating results, repeating our key-press data analyses while including individuals with poor No-go performance produced equivalent results: a significant reduction in key-pressing for female images for participants that previously inhibited females when compared to those that previously responded to females, *t*(74) = 2.98, *p* < 0.01, and no significant between-group differences in key-pressing for intact-male images or any of the scrambled images (all *p*s > 0.21).

It is noteworthy that both sexually appealing and non-appealing images received more negative hedonic evaluations after being inhibited – a finding consistent with prior evidence that inhibition affectively devalues both positively valenced and negatively valenced stimuli (Frischen et al., 2012). In contrast, only sexually appealing images showed an inhibition-related decrease in motivational incentive. On one hand, this finding is consistent with evidence that the processes determining hedonic value and motivational incentive may be relatively independent (e.g., Berridge and Robinson, 1995, 2003). On the other hand, the lack of an inhibition-related reduction in key-pressing for non-appealing images may reflect a floor effect produced by an overall lack of viewing incentive. Indeed the rate of key-pressing by our heterosexual male participants to view erotic-male images was so low, overall, that the “inhibit male” group could hardly have pressed less without knowing in advance which key was associated with each image type.

## General Discussion

Taken together, the results of two experiments demonstrate that inhibiting sexually appealing visual images not only leads to lower hedonic evaluations of these approach-related stimuli, but also decreases the motivational incentive to expend time and energy to see more of these images. Experiment 1 showed that both sexually appealing and non-appealing images were rated as less attractive when previously inhibited than when previously appearing as response targets, despite preferred-sex images being consistently rated as more attractive than images of participants’ non-preferred-sex. Experiment 2 established that inhibition has consequences for the motivational incentive of sex stimuli. Participants who had inhibited sexually appealing images were less likely to make repeated key-presses to see brief presentations of such stimuli than participants for whom sexually appealing stimuli previously appeared as response targets. Response inhibition therefore reduces the amount of time and effort expended to seek motivationally relevant stimuli. This suggests that the deleterious affective consequences of inhibition occur even for very motivationally salient stimuli and can lead to changes, not only in subjective emotional impressions, but also in the behavioral incentive to seek and obtain such items.

Taken together, our results support the view that, while there may be separate neural substrates for determining hedonic value and motivational incentive, response inhibition appears to impact both. When a prepotent response is inhibited, conflict may be detected by the dorsal anterior cingulate cortex (Bush et al., 2000). The anterior cingulate cortex, via connections to the orbitofrontal cortex and amygdala, may lead such affect-intensive regions to interpret the conflict and subsequent inhibition that is applied to resolve the conflict to be interpreted as a negative event. Electrophysiological evidence (Kiss et al., 2008) has revealed a negative-going event-related potential believed to originate in the ACC that peaks just after participants have viewed a cue to inhibit a motor response; the magnitude of this neural signal corresponds with the subsequent level of affective devaluation. Neural connections from the OFC/amygdala to the nucleus accumbens (Canteras et al., 1992) and mesotelencephalic dopamine system could likewise act as a substrate for cognitive inhibition to be translated both into an emotional devaluation and a decrease in motivational incentive.

Furthermore, our Experiment 2 results show that this reduction in motivational incentive carries over to different instances of the inhibited stimulus category. In other words, inhibiting many images from a given stimulus category may lead to a generalized reduction in motivational drive toward other items of that stimulus type. This finding extends prior observations that inhibition-related reductions in hedonic value can generalize to influence evaluations of other stimuli, as long as they are from the same category or otherwise share the same features as previously inhibited items (Raymond et al., 2003; Goolsby et al., 2009b). However, such effects, while general to a category of stimuli, may nevertheless still be tied to that specific class of stimuli. Indeed, while negative affective consequences are robust whenever a No-go cue forms an integral part of a stimulus (Frischen et al., 2012), they do not consistently generalize to other separate stimuli of different types that merely appear with a No-go cue (Veling et al., 2008).

Our evidence that inhibiting many images from a given stimulus category may lead to a generalized reduction in motivational drive toward other items of that stimulus type is particularly promising for the development of interventions aimed at selectively reducing the subjective appeal and motivational incentive of stimuli that trigger maladaptive behavior, such as drug-associated cues for substance abusers. The potential effectiveness of such an approach is underscored by recent work employing attentional inhibition to reduce attentional bias toward substance-related stimuli (e.g., Field and Eastwood, 2005; Fadardi and Cox, 2009; Field et al., 2009), and efforts to associate response inhibition with alcohol cues to reduce subsequent alcohol intake (Houben et al., 2011). Our finding that response inhibition can effectively reduce the amount of time and effort an individual is willing to expend to view erotic stimuli suggests that inhibition-based interventions may likewise be useful for reducing the motivational incentive of stimuli associated with other problematic behavior, such as pornography for the sexually compulsive (Cooper et al., 1999). Thus, taken together, our findings converge with other recent discoveries to support the possibility that the affective consequences of cognitive inhibition could be effectively harnessed for future clinical applications.

Previous work has demonstrated that the negative consequences of prior inhibition survive changes in tasks and types of stimuli, as well as unrelated intervening stimulus displays lasting at least 9 s (Fenske et al., 2005) and up to l8 s (Kiss et al., 2008; Frischen et al., 2012) – several seconds after being relevant to the task at hand. In the realm of cognitive processes, this reflects considerable persistence, similar to long-term negative priming (e.g., Grison et al., 2005). Investigating such effects over longer durations (hours, days, weeks) will be an important next step in assessing the therapeutic potential of tasks involving cognitive inhibition to reduce maladaptive motivational impulses.

## Conflict of Interest Statement

The authors declare that the research was conducted in the absence of any commercial or financial relationships that could be construed as a potential conflict of interest.
